# Incidence trends of squamous cell carcinoma of the head and neck (SCCHN) in the aging population––A SEER‐based analysis from 2000 to 2016

**DOI:** 10.1002/cam4.4134

**Published:** 2021-07-20

**Authors:** Melissa A. Taylor, Jeffery Switchenko, William Stokes, Mihir R. Patel, Mark McDonald, Conor Steuer, Ashley Aiken, Jonathan J. Beitler, Dong M. Shin, Nabil F. Saba

**Affiliations:** ^1^ Emory University School of Medicine Atlanta GA USA; ^2^ Department of Biostatistics and Bioinformatics Emory University Atlanta GA USA; ^3^ Emory University Winship Cancer Institute Atlanta GA USA

**Keywords:** elderly population, HPV, incidence trends, squamous cell carcinoma of the head and neck

## Abstract

**Background:**

Tobacco and alcohol use are risk factors for Squamous Cell Carcinoma of the Head and Neck (SCCHN); however, there is growing recognition of HPV as a risk factor for SCCHN. HPV‐related SCCHN is thought to affect mostly middle‐aged individuals but as the US population ages, it is important to evaluate the change in incidence of HPV‐ and non‐HPV‐related SCCHN in individuals who are ≥65 years old.

**Methods:**

This was a retrospective study using data from a population‐based cancer registry (SEER) to identify individuals ≥65 years old diagnosed with SCCHN between 2000 and 2016 also stratified by sex, race, and birth cohort. The subgroups of HPV‐associated and non‐HPV associated sites were analyzed independently. The incidence per year was calculated and joinpoint detection was used to identity significant changes in incidence trends and annual percent change (APC).

**Results:**

For HPV‐associated sites from 2000 to 2016, there was an average annual rate of 10.8 per 100,000 individuals with an APC of 2.92% (*p* = <0.05). For HPV‐ and non‐HPV‐related SCCHN males had a higher annual rate compared to females, 54.5 versus 18.0 in non‐HPV‐related and 19.1 versus 4.4 in HPV‐related sites. For non‐HPV‐related sites there was a decrease in APC across all stratified groups. For HPV‐related sites there was an increase in APC across all stratified groups, especially males (APC 8.82% 2006–2016 *p *< 0.05) and White individuals (APC 8.19% 2006–2016 *p *< 0.05). When stratified by birth cohort, HPV‐related SCCHN sites had a higher APC in ages 65–69 (8.38% *p* < 0.05) and 70–74 (8.54% *p* < 0.05).

**Conclusion:**

Among the population ≥65 years old from 2000 to 2016, the incidence rate for HPV‐related SCCHN sites has increased across all stratified groups, especially in White individuals, males, and age groups 65–74. The incidence rate for non‐HPV‐related sites has decreased across all stratified groups during this time.

## Graphical abstract

This study reports results that are consistent with other studies demonstrating that the prevalence HPV associated of head and neck cancer overall is increasing in the elderly population age ≥65 years. This increase is occurring when stratified by race, sex, and birth cohort however, the White male population is experiencing the most significant annual percent increase in the incidence of HPV‐associated head and neck cancers. HPV‐associated head and neck cancers are no longer a disease associated with younger patients and the increasing prevalence of this disease in the elderly population is important when considering the unique challenges of treatment for this age group.

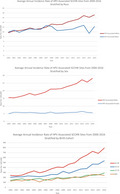

## 1 INTRODUCTION

The US population of those ≥65 years old is expected to double in size by 2060 from 49 million in 2016 to 95 million in 2060. By 2034, older adults will outnumber children for the first time in US history.^1^ The healthcare system will have to adjust to an aging population and the change in prevalence of disease, which includes adapting to the diagnosis and treatment of diseases that will become more prevalent in this population. Worldwide, there has been an overall decrease in incidence of head and neck cancers across all age groups and genders. However, for oropharyngeal cancer specifically, which is most closely associated with human papillomavirus (HPV) infection, the rate has been increasing by 2.5% per year from 2002 to 2012.^2^ From 2000 to 2012, there was a statistically significant increase in the incidence of HPV‐related head and neck cancers in people ≥65 compared with those <65 years old.^3^ Though HPV‐related oropharyngeal cancers affect a younger population than smoking‐ and alcohol‐related oropharyngeal cancers, the understanding of demographic changes in patients ≥65 years old is of increasing importance.

The epidemiology of head and neck cancers has changed drastically in the past decades in the United States.^4^ Major risk factors for the development of head and neck cancers include tobacco and alcohol use and infection with HPV.^5^ Over the past few decades in the United States, the prevalence of both of these risk factors has changed dramatically. The prevalence of tobacco abuse has decreased, with an annual percent decrease in tobacco use of 2% from 1980 to 2012.^6^ Meanwhile, the prevalence of HPV has increased from 1984 to 2004 among men and White patients and among patients aged 50–59 years old.^7^ A meta‐analysis found an increase in HPV‐related oropharyngeal squamous cell carcinomas from 20% in 1990 to 65% in 2000.^8^

It is important to consider the impact of the increasing prevalence of HPV‐related head and neck cancers in patients who are ≥65 years old because they represent a historically unique population in head and neck cancers. These patients have a higher prevalence of comorbidities at the time of diagnosis and they are more likely to develop treatment‐related comorbidities in the short term, such as pneumonia, weight loss, dysphagia, malnutrition, and dental issues, and in the long term >5 years after diagnosis such as weight loss, dysphagia, new hypertension, and anemia.^9^ Older patients with head and neck cancers are also more likely to suffer from depression and to be hospitalized.^10^ Optimal management of this patient population is challenging as they are underrepresented in clinical trials and may not derive the same benefit from systemic agents (e.g., cetuximab and cisplatin) compared to a younger population.^11^, ^12^, ^13^ In Canada, for example, concurrent cisplatin is not generally offered to patients ≥70 years old. Older patients experience significant treatment‐related toxicities with chemotherapy and radiation compared to radiation alone.^14^ Many patients with head and neck cancers present at more advanced stages (stage III or IV) and require multimodal therapy with surgery, chemotherapy, and radiation. Since older patients are more likely to have comorbidities at diagnosis, multimodal treatment can be challenging and places them at higher risk for developing treatment‐related toxicities.^15^ Due to the aging population in the United States and changes in trends of head and neck cancers it is essential to understand the incidence trends of head and neck cancers in this population.

## 2 METHODS

Demographic characteristics and cancer incidence data were obtained from the SEER‐18 + Hurricane Katrina Impacted Louisiana Cases (Surveillance, Epidemiology, and End Results) database spanning 2000–2016. SEER‐18 is derived from 18 cancer registries in the United States and covers 27.8% of the cancer cases in the United States (http://seer.cancer.gov). This study looked specifically at older adults defined as age ≥65 years at the time of diagnosis. The study population was stratified by race (Black or White) and sex (male or female) and birth cohort (age 65–69, age 70–74, age 75–79, and age 80+) for subgroup analysis (Table 1). Anatomic sites for head and neck cancer sites not traditionally associated with HPV were identified based on their ICD‐0–3 site codes arising from tongue (C020–C023, C028–C029), lip (C000–C009), floor of mouth (C040–C049), nasopharynx (C110–C119), hypopharynx (C129–C139), gum and other mouth (C030–C039, C040–C049, C050–C059, C060–C069), larynx (C320–C329), and other oral cavity (C140, C148). Anatomic sites associated with HPV+head and neck cancers were also analyzed separately. These consisted of base of tongue (C019), tonsils (C024, C090, C091, C098, C099), vallecula (C100), oropharynx (C101–104, C108, C109), and Waldeyer's ring (C142). Histological site codes were used to identify squamous cell carcinoma (8050–8076, 8078, 8083–8084, 8094). Cancer cases with malignant behavior code (3 for behavior code) were included and benign cases (0 or 1 for behavior code) were excluded. Although HPV status is available in SEER Stat, the data are limited to only years 2010–2016. Additionally, only proportions of HPV cases are reported rather than incidence, resulting in a large number of cases with missing data. Due to the limited availability of HPV status in the SEER dataset, sites highly associated with HPV status were used to indicate incidence of HPV in this population.

**TABLE 1:** Demographics of the study population for squamous cell carcinoma of the head and neck age ≥65 (2000–2016)

	HPV related	Non‐HPV related
Total	19,106	58,635
Demographic		
Male	14,688 (76.9%)	40,588 (69.2%)
Female	4,418 (23.1%)	18,047 (30.8%)
White	16,725 (87.5%)	50,047 (85.4%)
Black	1,586 (8.3%)	5,085 (8.7%)
Birth cohort		
65–69	7,556 (39.6%)	16,321 (27.8%)
70–74	5,207 (27.3%)	14,496 (24.7%)
75–79	3,290 (17.2%)	11,896 (20.3%)
80+	3,053 (15.9%)	15,922 (27.2%)

Statistical analyses were performed using SEER*Stat, version 8.3.9, and Joinpoint regression program, version 4.7.0.0. Age‐adjusted incidence rates expressed per 100,000 persons were calculated as the weighted average of crude cancer rates using the population from the 2000 US census as the standard population. Annual percent change (APC) was calculated using the Joinpoint regression program using log‐linear regression and comparison of the incidence trends.^16^ Two‐sided *p* levels at the alpha = 0.05 level were considered to be statistically significant.

## 3 RESULTS

**FIGURE 1:**
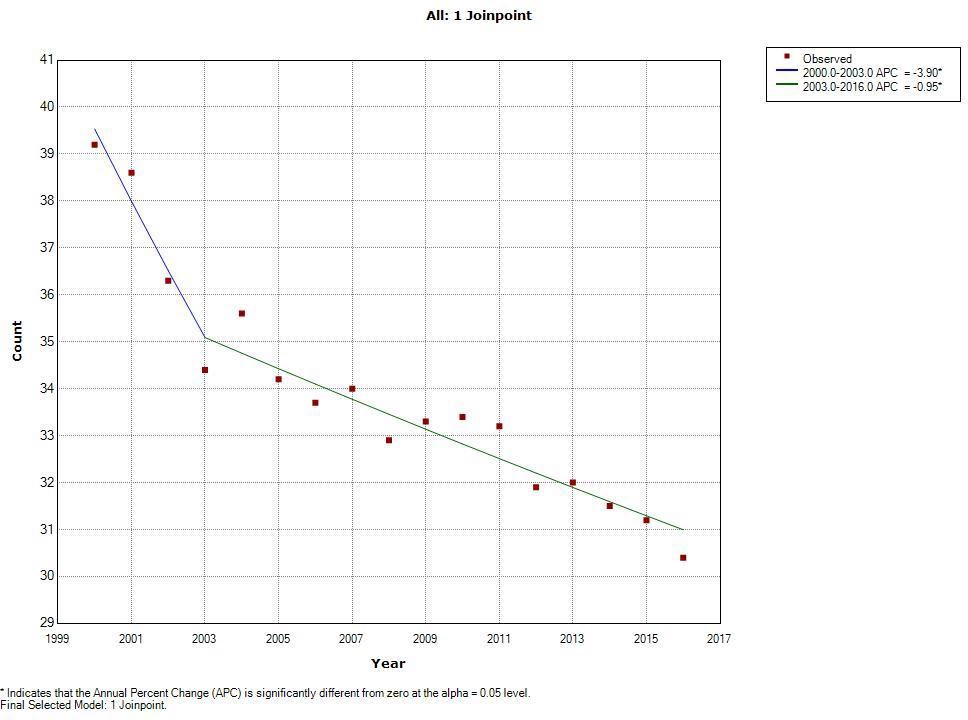
This figure shows the joinpoint analysis of non‐HPV related sites. The line represents the model crude rate and the red dots represent the observed crude rate. The slope of the line is the APC (annual percent change) in incidence

**FIGURE 2:**
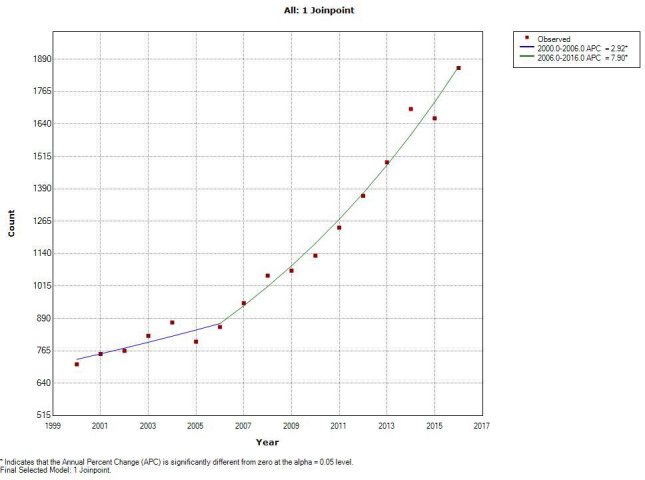
This figure shows the joinpoint analysis of HPV related sites. The line represents the model crude rate and the red dots represent the observed crude rate. The slope of the line is the APC (annual percent change) in incidence

Among individuals’ ≥65 years old, the average annual incidence rate for Non‐HPV‐related sites (Table 2) was 33.6 (CI 33.3–33.9) per 100,000 persons from 2000 to 2016. There was a joinpoint at 2003 with a statistically significant APC from 2000 to 2003 of −3.90% (*p* < 0.05) per year and from 2003 to 2016 of −0.95% per year (Figure 1). For HPV‐related sites (Table 3) the average annual incidence rate was 10.8 per 100,000 persons (CI 10.7–11.0). There was a joinpoint at 2006 with a statistically significant APC from 2000 to 2006 of 2.92% per year and an even greater increase from 2006 to 2016 of 7.90% per year (Figure 2).

**TABLE 2:** Average annual crude SCC head and neck cancers incidence rates and trends Non‐HPV‐related sites (2000–2016)

Non‐HPV‐related sites	Average annual rate (per 100,000)	Slope 1		Slope 2	
		Years	APC	Years	APC
Total	33.6 (33.3–33.9)	2000–2003	−3.90*	2003–2016	−0.95*
Sex					
Males	54.5 (54.0–55.1)	2000–2003	−4.29*	2003–2016	−1.27
Females	18.0 (17.7–18.3)	2000–2016	−1.09*		
Race					
Black	33.5 (32.6–34.4)	2000–2016	−2.33		
White	34.9 (34.6–35.2)	2000–2003	−3.52	2003–2016	−0.85*
Sex and Race					
White males	55.8 (55.2–56.4)	2000–2003	−1.22*	2003–2016	−1.22*
White females	19.1 (18.8–19.4)	2000–2016	−1.02*		
Black males	64.0 (62.0–66.2)	2000–2016	−3.07*		
Black females	13.6 (12.9–14.4)	2000–2016	−0.97		

* Indicates that the annual percent change (APC) is significantly different from zero at the alpha = 0.05 level.

**TABLE 3:** Average annual crude SCC head and neck cancers incidence rates and trends HPV‐related sites (2000–2016)

HPV‐related sites	Average annual rate (per 100,000)	Slope 1	Slope 2		
		Years	APC	Years	APC
Total	10.8 (10.7–11.0)	2000–2006	2.92*	2006–2016	7.90*
Sex					
Males	19.1 (18.8–19.4)	2000–2006	4.53*	2006–2016	8.82*
Females	4.4 (4.3–4.6)	2000–2007	−0.34	2007–2016	4.91*
Race					
Black	10.2 (9.7–10.7)	2000–2016	4.15*		
White	11.6 (11.4–11.8)	2000–2006	2.55	2006–2016	8.19*
Sex and Race					
White males	20.2 (19.9–20.6)	2000–2006	4.20*	2006–2016	9.20*
White females	4.8 (4.6–4.9)	2000–2016	2.68*		
Black males	19.8 (18.7–20.9)	2000–2016	4.78*		
Black females	3.8 (3.4–4.2)	2000–2016	2.76*		

* Indicates that the annual percent change (APC) is significantly different from zero at the alpha = 0.05 level.

When the cohort was stratified by sex the rate of both HPV‐related and non‐related SCCHN was greater among males compared to females. For Non‐HPV‐related sites the rate among males was 54.5 per 100,000 (CI 54.0–55.1) compared to 18.0 per 100,000 in females (CI 17.7–18.3). The APC decreased for both males and females from 2000 to 2016. For males there was a joinpoint in 2003 with a statistically significant APC of −4.29% from 2000 to 2003 and a non‐significant APC of −1.27% from 2003 to 20016. For females the APC from 2000 to 2016 was −1.09% (*p* < 0.05). For HPV‐related sites the opposite was observed with an increase in APC from 2000 to 2016 for males and females. For males the average annual rate was 19.1 per 100,000 (CI 18.8–19.4) compared to 4.4 per 100,000 (CI 4.3–4.6) in females. For males there was a joinpoint in 2006 with an APC of 4.53% (*p* < 0.05) from 2000 to 2006 and 8.82% (*p *< 0.05) from 2006 to 2016. For females there was a joinpoint in 2007 with a statistically significant APC of 4.91% from 2007 to 2016.

When the cohort was stratified by race the average annual crude rate was similar between White and Black individuals but the trends in APC varied. For non‐HPV‐related sites Black individuals had an average annual rate of 34.9 per 100,000 persons compared to 33.5 in White individuals. The APC for both White and Black individuals decreased from 2000 to 2016 with APC −2.33% for Black individuals and −3.52% from 2000 to 2003 and −0.85% from 2003 to 20016 for White individuals. For HPV‐related sites the average annual rate was 10.2 for Black individuals and 11.6 for White individuals. The APC was increased from 2000 to 2016 for both Black and White individuals with 4.15% (*p *< 0.05) for Black and 2.55% (*p *> 0.05) from 2000 to 2006 and 8.19% (*p *< 0.05) for White individuals.

The cohort was further stratified into birth cohorts, age 65–69, age 70–74, age 75–79, and age ≥80 (Table 4). For non‐HPV‐related sites all birth cohorts had an average annual incidence rate of >30 per 100,000 with the greatest rate in cohort age 75–79 of 36.4 (CI 35.7–37.0). The APCs in rate were decreased across all birth cohorts for non‐HPV‐related SCCHN. For cohorts 65–69 and 75–79 there was a joinpoint in 2003 with a large decrease in APC from 2000 to 2003 compared to 2003–2016. From 2000 to 2003 the APC for ages 65–69 was −5.71% (*p *< 0.05) and −5.27% (*p *< 0.05) for ages 75–79. The APC decreased from 2003 to 2016 with −1.68% (*p *< 0.05) for ages 65–69 and −0.33% for ages 75–79 which was non‐significant. For HPV‐related sites the average annual rate decreased as age increased with the greatest rate in age 65–69 of 14.2 (CI 13.9–14.5) and smallest in age 75–79 of 10.7 (CI 9.7–10.5). For all birth cohorts the APC was positive. For age 65–69 the APC was 8.38% from 2000 to 2016 (*p *< 0.05) and for age 70–74 there was a joinpoint in 2006 with a significant APC of 8.54% from 2006 to 2016 (*p *< 0.05). The later birth cohorts still had a positive APC, however, it was not as great as the younger birth cohorts. From 2000 to 2016 the APC was 4.08% for age 75–79 (*p *< 0.05) and 4.62% for age ≥80 (*p *< 0.05).

**TABLE 4:** Average Annual Crude SCC Head and Neck Cancers Incidence Rates and Trends Stratified by Birth Cohort (2000–2016)

Birth cohort	Average annual rate (per 100,000)	Slope 1		Slope 2	
		Years	APC	Years	APC
Non‐HPV related					
Age 65–69	30.6 (30.1–31.1)	2000–2003	−5.71*	2003–2016	−1.68*
Age 70–74	35.3 (34.6–35.8)	2000–2016	−1.64*		
Age 75–79	36.4 (35.7–37.0)	2000–2003	−5.27*	2003–2016	−0.33
Age 80+	33.5 (33.0–34.0)	2000–2016	−0.35		
HPV related					
Age 65–69	14.2 (13.9–14.5)	2000–2016	8.38*		
Age 70–74	12.6 (12.3–13.0)	2000–2006	1.51	2006–2016	8.54*
Age 75–79	10.1 (9.7–10.4)	2000–2016	4.08*		
Age 80+	11.7 (11.2–12.3)	2000–2016	4.62*		

* Indicates that the annual percent change (APC) is significantly different from zero at the alpha = 0.05 level.

## 4 DISCUSSION

This study demonstrates that the APC in incidence rate is decreasing for non‐HPV‐associated squamous cell carcinomas of the head and neck when stratified by gender and race and also by birth cohort. Every birth cohort experienced a negative APC of the incidence rate which was most pronounced in birth cohort aged 65–69. SCCHN in HPV‐associated locations was found to have an increase in the APC of the incidence rate over the same time period. Males had a larger annual incidence rate compared to females by just over 4x as much as females. The overall positive APC from 2006 to 2016 appears to be mostly driven by White males suggesting that this demographic is experiencing the greatest increase in disease incidence.

When the population was stratified by birth cohort, those in the 65‐ to 69‐year‐old and 70‐ to 74‐year‐old groups had the highest average annual incidence and APC when compared to those aged 75–79 and 80+. The trends are consistent with the study by Tota et al. from 2019 which forecasted that the incidence of oropharynx cancer would increase dramatically from 2016 to 2029 in individuals from 65 to 74 years of age.^17^ The increase was most dramatic in White males with an EAPC of 3.8% from 1992 to 2015, which is consistent to what was observed in this analysis. This study also showed an increase in APC among White women which is consistent with the Tota et al. study, however, Black women and Black men were also found to have an increase in the APC, respectively, was not consistent with the Tota et al. study. In each birth cohort White men had an increase in APC but the rate decreased as the cohorts increase in age. The results of this study are consistent with recent studies^3^, ^17^, ^18^, ^19^, ^20^ demonstrating that the incidence rate for HPV‐related sites of SCCHN is increasing dramatically among those ≥65 years old. This increase is most pronounced in the White male demographic, however, the APC for White females, Black males, and Black females is also positive from 2000 to 2016 indicating an increase in incidence of HPV‐related SCCHN among these demographic groups.

The reasons for this increase remain unclear due to the limitations of the database to quantify tobacco or alcohol use, sexual behavior, or other risk factors. One possibility is that this could be due to increased awareness of the association between HPV and HNC, however, a Swedish study looked at banked oropharyngeal tumor pathology samples from the 1970s onward and found that only 28% of these tumors were associated with HPV in the 1970s, which increased to 77% in the 2000s and 93% in the 2010s.^21^ Another possibility is the change in social risk factors, such as sexual behavior among this cohort. Studies have shown that number of sexual partners and HSV‐2 prevalence has decreased following the birth cohort of those age 60 or younger.^22^, ^23^

The variation between men and women is also an interesting finding in this study. It is unclear why men have such a higher rate of HPV‐related cancers when compared to women; however, as in the studies mentioned above, changes in sexual practices could be contributing to this difference. A 2017 study in the Journal of Infectious Diseases by Lewis et al. demonstrated that the prevalence of HPV was significantly higher in males compared to females in cohorts 40–49 years old and 50–59 years old.^24^ Another study published in JAMA in 2010 by Gillison et al. showed that among men and women age 14–69 in the United States, the overall prevalence of oral HPV infection was three times more common in men compared to women.^25^ These findings are consistent with the results of this study which found an average annual rate of HPV‐related sites of SCCHN to be 19.1 per 100,000 in males compared to 4.4 per 100,000 in females.

There is also a differing annual rate of HPV‐associated SCCHN when stratified by race. White individuals had the highest APC from 2006 to 2016 compared to Black individuals. This pattern has been observed in multiple other studies^17^, ^26^, ^27^ with an increase in incidence among White individuals compared to Black individuals. As the population ages there may be a shift between the difference in prevalence between White and Black individuals. Studies looking at the prevalence of oral HPV infections show that there is an increase in overall oral HPV infections in Black individuals compared to White individuals.^28^, ^29^ This suggests that as the population continues to age, if this trend in prevalence is maintained then there will be an increase in rate of HPV‐associated SCCHN among Black individuals. This is an important consideration not only because of changes in at‐risk demographics but because of outcomes for HPV‐associated SCCHN. A 2016 study examining racial disparities between head and neck cancer types found that White patients had a higher incidence of HPV‐associated cancers compared to Black patients, however, Black patients were more likely to present with more advanced non‐HPV‐associated SCCHN cancers and had overall decreased survival compared to White patients.^30^ A study by Jiron et al. found that histologic samples from oropharyngeal sites of black individuals were less likely positive for HPV and this trend held when smoking and alcohol use was controlled for. Since HPV associated cancers tend to have more favorable outcomes when compared to non‐HPV associated cancers this could account for some of the disparities in survival. Other social and behavioral disparities need to be further examined to account for this difference in survival.^31^

The results of the study are important to demonstrate that the epidemiology of head and neck cancers among elderly patients is changing. The risk factor profile is changing with the decrease in tobacco resulting in a decline in non‐HPV‐related head and neck cancers and a change in sexual practices resulting in an increase in the incidence of HPV infection and HPV‐associated head and neck cancers. Older patients are often under‐represented in clinical trials due to their age and higher prevalence of comorbidities. Older adults with head and neck cancers have a higher risk of functional and cognitive impairment, depressive symptoms, and social isolation, which are all important factors when considering treatment plans for patients.^32^ Older patients with head and neck cancers are also more likely to have additional comorbidities and to develop treatment‐related complications thus resulting in poorer survival outcomes compared to younger patients.^9^ Elderly patients also have been shown to have difficulty tolerating intensification strategies for treatment such as chemotherapy and radiation in addition to surgery or radiation alone and although these strategies have shown improved survival in younger populations, they have not shown survival benefit in older patients.^3^

The study had a number of limitations. In the SEER database, HPV status is limited to years 2010–2016 and only proportions of HPV+cases are reported rather than incidence rates of cases and as result a large number of cases are missing data and HPV status. Therefore, site‐specific codes of anatomical sites for SCCHN highly associated with HPV were used as a proxy for HPV‐associated cancers. Due to this limitation, assumption of HPV status is made by the location of the tumor and thus, there are likely HPV‐associated tumor cases that are not represented in the data and tumors that are not HPV‐associated that are represented in the HPV‐associated case count based on anatomical location. Additionally, there is not a way to stratify based on risk factors such as tobacco or alcohol use or sexual practices; so the reasons for the change in incidence are based on assumption of known risk factors from prior study data. This study also did not address stage of disease at diagnosis or changes in survival outcomes, which are important considerations to examine when determining the overall effect the change in prevalence or incidence of SCCHN.

## 5 CONCLUSION

This study reports results that are consistent with other studies^3^, ^17^ demonstrating that the prevalence of head and neck cancers overall is decreasing in the elderly population age ≥65 years, however, the incidence of HPV‐associated head and neck cancers is increasing among this age group. The White male population is experiencing the most significant annual percent increase in the incidence of HPV‐associated head and neck cancers. This study supports the importance of including older patients in clinical trials for the treatment of HPV‐associated SCCHN given the demonstrated increase in APC of the incidence rate. HPV‐associated head and neck cancers are no longer a disease associated with younger patients and the increasing prevalence of this disease in the elderly population is important when considering the unique challenges of treatment for this age group.

## CONFLICT OF INTEREST

None.

## ETHICAL STATEMENT

Ethical approval was not sought for the study.

## Data Availability

Data are available through SEER database.
